# Tobramycin reduces key virulence determinants in the proteome of *Pseudomonas aeruginosa* outer membrane vesicles

**DOI:** 10.1371/journal.pone.0211290

**Published:** 2019-01-25

**Authors:** Katja Koeppen, Roxanna Barnaby, Angelyca A. Jackson, Scott A. Gerber, Deborah A. Hogan, Bruce A. Stanton

**Affiliations:** 1 Department of Microbiology and Immunology, The Geisel School of Medicine at Dartmouth, Hanover, New Hampshire, United States of America; 2 Department of Molecular and Systems Biology, The Geisel School of Medicine at Dartmouth, Hanover, New Hampshire, United States of America; Universidad Nacional de la Plata, ARGENTINA

## Abstract

Tobramycin is commonly used to treat *Pseudomonas aeruginosa* lung infections in patients with Cystic Fibrosis (CF). Tobramycin treatment leads to increased lung function and fewer clinical exacerbations in CF patients, and modestly reduces the density of *P*. *aeruginosa* in the lungs. *P*. *aeruginosa* resides primarily in the mucus overlying lung epithelial cells and secretes outer membrane vesicles (OMVs) that diffuse through the mucus and fuse with airway epithelial cells, thus delivering virulence factors into the cytoplasm that modify the innate immune response. The goal of this study was to test the hypothesis that Tobramycin reduces the abundance of virulence factors in OMVs secreted by *P*. *aeruginosa*. Characterization of the proteome of OMVs isolated from control or Tobramycin-exposed *P*. *aeruginosa* strain PAO1 revealed that Tobramycin reduced several OMV-associated virulence determinants, including AprA, an alkaline protease that enhances *P*. *aeruginosa* survival in the lung, and is predicted to contribute to the inhibitory effect of *P*. *aeruginosa* on Phe508del-CFTR Cl^-^ secretion by primary human bronchial epithelial cells. Deletion of the gene encoding AprA reduced the inhibitory effect of *P*. *aeruginosa* on Phe508del-CFTR Cl^-^ secretion. Moreover, as predicted by our proteomic analysis, OMVs isolated from Tobramycin treated *P*. *aeruginosa* had a diminished inhibitory effect on Phe508del-CFTR Cl^-^ secretion compared to OMVs isolated from control *P*. *aeruginosa*. Taken together, our proteomic analysis of OMVs and biological validation suggest that Tobramycin may improve lung function in CF patients infected with *P*. *aeruginosa* by reducing several key virulence factors in OMVs that reduce CFTR Cl^-^ secretion, which is essential for bacterial clearance from the lungs.

## Introduction

*Pseudomonas aeruginosa* is a Gram-negative, opportunistic pathogen that is common in immunocompromised individuals, and chronically infects the lungs of many individuals with chronic obstructive pulmonary disease, ventilator-associated pneumonia, and cystic fibrosis (CF) [1, 2]. In CF, the most common lethal genetic disease in Caucasians, chronic lung infection is dominated by *P*. *aeruginosa*, which is a major cause of lung function decline, morbidity and mortality [1, 3–6]. In the lung, *P*. *aeruginosa* stimulates the secretion of pro-inflammatory cytokines by airway epithelial cells, including IL-6 and IL-8, which promote the migration of macrophages and neutrophils into the lung. These recruited phagocytes kill bacteria, thereby clearing the infection in healthy (non-CF) individuals [3–5]. However, mutations in the *CFTR* gene cause numerous defects in the innate immune response leading to chronic lung infections [1, 3–6]. *P*. *aeruginosa* also reduces wt-CFTR as well as VX-809 and VRT-325 stimulated Phe508del-CFTR Cl^-^ secretion by airway epithelial cells, an effect that reduces mucociliary clearance of bacteria [1, 7–14].

To establish and maintain lung infections in CF, *P*. *aeruginosa* secretes many virulence-related factors that subvert the host innate immune response [1, 2]. For example, *P*. *aeruginosa* secretes rhamnolipids that promote ciliastasis, and alginate, which increases mucus production by goblet cells, thereby reducing immune recognition and mucociliary clearance of bacteria from the lungs [1, 2, 15]. Ciliary beating and mucociliary clearance are also reduced by pyocyanin, which decreases CFTR Cl^-^ secretion by airway epithelial cells [16, 17]. Phospholipase C (PlcH), β-lactamase, CFTR Inhibitory Factor (Cif) and LasB also inhibit CFTR Cl^-^ secretion by airway epithelia cells [1, 18]. By contrast, *P*. *aeruginosa* also secretes virulence factors that stimulate CFTR Cl^-^ secretion including LPS, homoserine lactone and flagellin (reviewed in [1, 2]). Other known *P*. *aeruginosa* virulence factors include AprA and proteins from the Alp operon [19]. The alkaline protease AprA is cytotoxic to host cells and suppresses the cellular and humoral immune response of the host [20]. AprA has also been shown to prevent complement-mediated phagocytosis [21]. The lysis phenotype activator protein AlpA as well as downstream effectors AlpD and AlpE are associated with *P*. *aeruginosa* self-lysis, a mechanism of programmed cell death that promotes virulence and lung colonization by surviving *P*. *aeruginosa* [19]. *P*. *aeruginosa* deletion mutants for AlpA and AlpBCDE have attenuated virulence and reduced lung colonization in a murine infection model [19].

In histological analyses of explanted lungs from individuals with CF, *P*. *aeruginosa* is found primarily in the mucus layer overlying lung epithelial cells. *P*. *aeruginosa* secretes outer membrane vesicles (OMVs), spheroid buds of the outer membrane 10 to 300 nm in diameter [22], that diffuse through the mucus layer and fuse with lipid rafts in the apical plasma membrane of airway epithelial cells, thereby delivering virulence factors and sRNAs into the cytoplasm of lung epithelial cells [7, 8, 22–25].

When *P*. *aeruginosa* infects the lungs of CF patients, they are treated with antibiotics, primarily Tobramycin, to suppress infection, reduce pulmonary exacerbations, and minimize the decrease in lung function [26–29]. Nebulized Tobramycin inhalation solution (TIS) is administered in cycles of 28 days on drug followed by 28 days off drug. Although it increases lung function (measured as forced expiratory volume in one second, FEV_1_) and reduces mortality in CF patients [30, 31], TIS has only a modest effect on the burden of *P*. *aeruginosa* in the lungs [27–29]. These observations led to the suggestion that some of the clinical benefit of Tobramycin may be related to anti-inflammatory effects and/or a reduction in the production of virulence factors by *P*. *aeruginosa* [29]. Accordingly, the goal of this project was to test the hypothesis that Tobramycin reduces the abundance of virulence factors in OMVs secreted by *P*. *aeruginosa*. To this end, we isolated secreted OMVs from *P*. *aeruginosa* strain PAO1 grown with a sub-inhibitory concentration of Tobramycin (1 μg/ml), or untreated controls, and used a liquid chromatography—tandem mass spectrometer (LC-MS/MS) approach to examine the effect of Tobramycin on the protein content of OMVs. We found that Tobramycin decreased the abundance of several virulence-related proteins, including AprA, in OMVs, thereby reducing the ability of OMVs to inhibit Phe508del-CFTR Cl^-^ secretion by human bronchial epithelial cells, an effect that may improve clearance of *P*. *aeruginosa* from the lungs.

## Materials and methods

### *Pseudomonas aeruginosa* strains

*P*. *aeruginosa* strain PAO1 was grown in lysogeny broth (LB, Invitrogen, Grand Island, NY) in the presence or absence of Tobramycin (1 μg/ml, Sigma, St. Louis, MO) as described [32–37]. This sub-inhibitory concentration was chosen because it did not affect growth rate or yield in PAO1 under these conditions. An *aprA* deletion mutant in *P*. *aeruginosa* strain PA14 was generated using previously published methods [38].

### Outer membrane vesicle (OMV) isolation

OMVs from three separate overnight cultures of *P*. *aeruginosa* grown in LB (Ctl OMVs) or in LB with Tobramycin (Tobi OMVs) were isolated and purified by Optiprep gradient ultracentrifugation as described in detail in [23]. The gradient purification separates OMVs from non-OMV-associated protein complexes, like ribosomes or flagella, that may co-sediment with OMVs during the initial centrifugation step [8, 39]. OMVs were quantified using nanoparticle tracking analysis (NanoSight NTA, Malvern Panalytical) and OMV protein content was measured using a Micro BCA Protein Assay Kit (Thermo Scientific, Rockford, IL).

### Proteomic identification of proteins in OMVs

Proteins from OMVs (20 μg per sample) were precipitated using TCA exactly as described previously [40]. Precipitated proteins were digested using trypsin (1:100 w/w) at 37°C overnight, followed by LC-MS/MS analysis, database searching and curation exactly as described previously [24]. Peptide quantification was performed using MassCroQ [41] and protein abundances were estimated using the iBAQ approach as described [42]. Sample-specific iBAQ values were corrected for total abundance by normalization prior to reporting. This label-free approach allows for the comparison of the abundance of different proteins in a sample. Raw and normalized proteomic data are provided in S1 Table. Full raw proteomics data have been submitted to the ProteomeXchange Consortium (accession number PXD012071).

### Cell culture

Primary CF human bronchial epithelial (CF-HBE) cells from three donors homozygous for the Phe508del mutation in CFTR were obtained from Dr. Scott Randell from the University of North Carolina and cultured as described previously [43]. Prior to measurements of Phe508del CFTR Cl^-^ secretion, cells were grown as a monolayer in air-liquid interface culture for 3–4 weeks as described [9]. CFBE41o- cells homozygous for the Phe508del CFTR mutation and stably expressing Phe508del CFTR (CFBE cells) were a gift of Dr. J. P. Clancy, University of Cincinnati, and grown in culture for 7–10 days as described [44].

### Measurements of Phe508del CFTR Cl- secretion

CF-HBE and CFBE cells were treated with VX-809 (3 μM, Selleckchem, Houston, TX) for 48 h to increase Phe508del CFTR Cl^-^ secretion. VX-809 is a key component of Orkambi, an FDA drug approved for use in CF patients to stimulate Phe508del CFTR Cl^-^ secretion by CF-HBE cells. To provide biological validation for our proteomics analysis, in one set of experiments, the same number of OMVs isolated from control or Tobramycin treated *P*. *aeruginosa* were added to the apical side of CF-HBE or CFBE cells for 1.5 h before measurements of Phe508del CFTR Cl^-^ secretion. OMVs were quantified using FM 4–64 fluorescent dye (Invitrogen) as described previously [25]. In a second validation experiment either *P*. *aeruginosa* (PA14-wt) or PA14, in which the *aprA* gene was deleted (PA14-Δ*aprA*), was added to the apical side of CF-HBE cells at a multiplicity of infection (MOI) of 30:1 for 6 hours. Phe508del CFTR Cl^-^ secretion was measured as described in detail previously [9].

### Data analysis and statistics

Raw integrated peak data (iBAQ) were normalized to yield the same total abundance for each sample. QPROT [45] with standard parameters was used for differential protein abundance analysis of normalized data for 761 high confidence proteins that were detected in at least two replicate samples. Proteins with FDR < 0.05 were considered significantly differentially abundant in Tobi OMVs. All other data analysis and visualization of results was performed with the R language and environment for statistical computing version 3.4.3 [46]. 757 high confidence proteins detected in PAO1 Ctl OMVs were compared to PAO1 OMV proteins identified in previous proteomic studies [47–50]. We also compared proteins detected in PAO1 Ctl OMV in this study with proteins in OMVs secreted by *P*. *aeruginosa* strain PA14 and two clinical isolates of *P*. *aeruginosa* [25]. UniProtKB accession numbers were converted to *Pseudomonas* gene identifiers using the UniProt ID mapping tool at http://www.uniprot.org/ [51]. Venn diagrams were created using the R package “VennDiagram” [52]. Protein subcellular localization annotations were obtained from the *Pseudomonas* Genome Database at http://www.pseudomonas.com/ [53]. Phe508del CFTR Cl^-^ currents were analyzed in GraphPad Prism version 6.0h using repeated measures ANOVA.

## Results and discussion

### Control OMV core proteome

**Fig 1:**
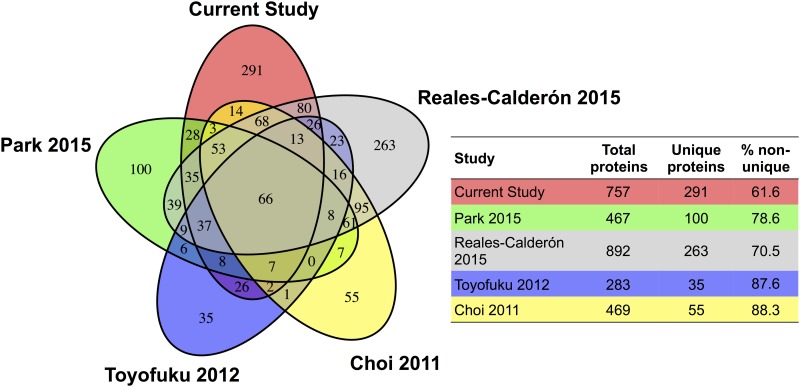
Core proteome of OMVs isolated from control *P*. *aeruginosa*. We identified a total of 757 proteins in OMVs secreted by planktonic *P*. *aeruginosa* (PAO1). Of these, 466 proteins (62%) were detected in OMVs secreted by planktonic *P*. *aeruginosa* in at least one other published study, and 291 proteins were unique to the present study. The core proteome, defined as proteins detected in OMVs secreted by planktonic *P*. *aeruginosa* in this and all four previous studies [47–50], was composed of 66 proteins.

We identified 757 proteins in OMVs secreted by planktonic *P*. *aeruginosa* strain PAO1 (Fig 1). Of these, 466 proteins (62%) were detected in OMVs secreted by planktonic *P*. *aeruginosa* in at least one of four published studies on PAO1 OMVs using a similar approach [47–50], and 291 proteins were unique to the present study. Previous studies on OMVs secreted by PAO1 have identified as few as 283 proteins and as many as 892 proteins, thus, the identification of 757 proteins in this study is within the published range [47–50]. The core proteome, which we define herein as proteins detected in OMVs secreted by planktonic *P*. *aeruginosa* (PAO1) in this and all four previous studies, was composed of only 66 proteins (Fig 1).

**Fig 2:**
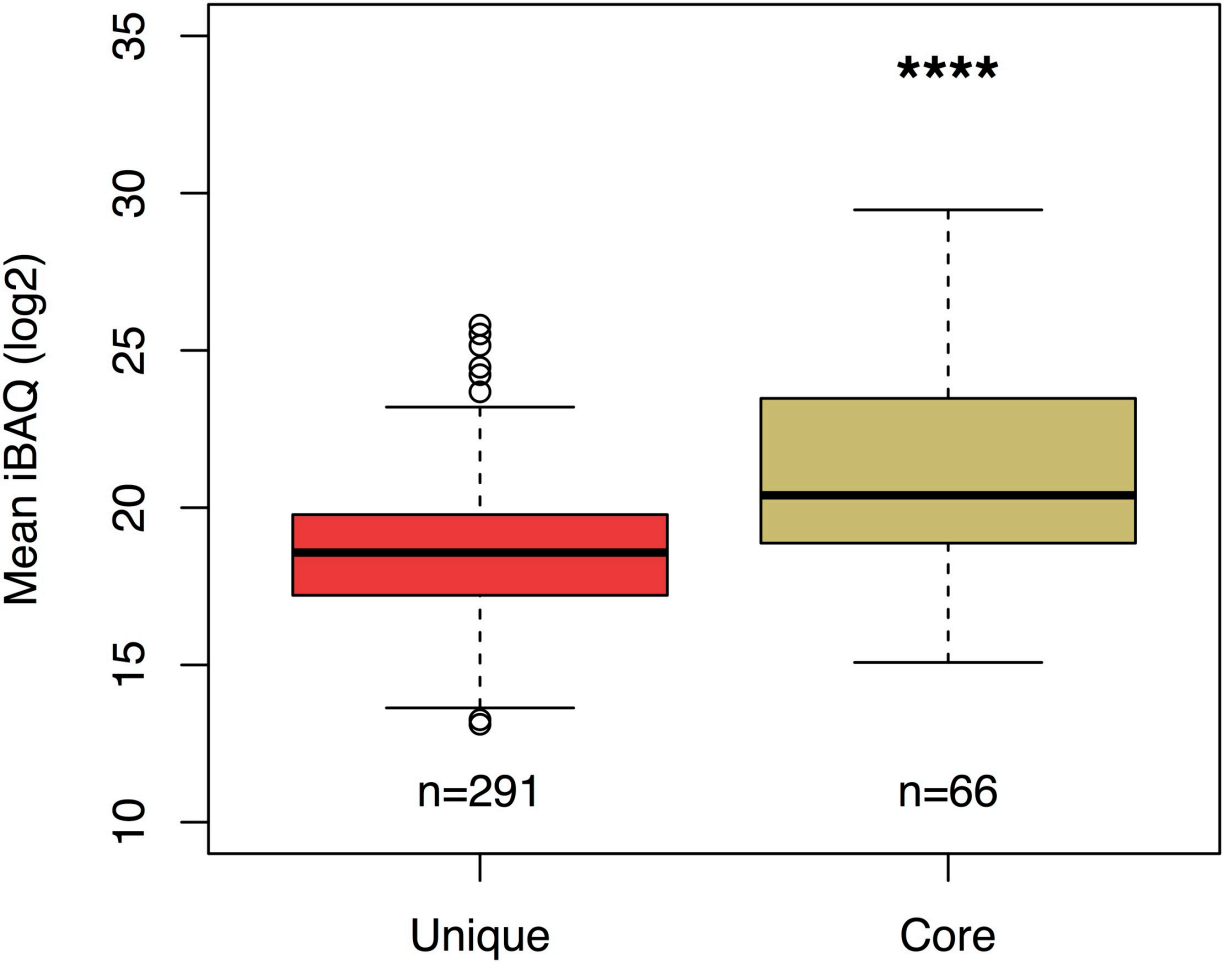
Mean log2 iBAQ of unique and core proteins. PAO1 OMV core proteins are significantly more abundant than unique proteins. ****P<0.0001 versus unique.

In our study, core proteins were significantly more abundant than the unique proteins identified (Fig 2). The low number of core proteins is likely due a variety of factors including strain differences in PAO1 among laboratories, different growth conditions in the studies, nutrient availability in the incubation media, different amounts of input protein and differences in mass spectrometry methods and analysis [47–50].

### Subcellular localization of the core proteome

Interrogation of the *Pseudomonas* Genome Database revealed that the 66 proteins in the core proteome were localized to four major categories: (1) Outer Membrane Vesicle, (2) Outer Membrane, (3) Cytoplasmic Membrane, and (4) Cytoplasmic (Table 1). This observation agrees with the subcellular localization of PAO1 OMV proteins reported in previous studies [47–50]. While the membrane vesicles produced by Gram-negative bacteria are commonly referred to as outer membrane vesicles, new evidence suggests that Gram-negative bacteria also produce vesicles that contain outer as well as inner membrane components [54]. The latter have recently been designated as Outer-inner membrane vesicles (O-IMVs) and have been found to constitute about 0.5% of the total membrane vesicle population in *P*. *aeruginosa* strain PAO1 [55]. The presence of cytoplasmic and cytoplasmic membrane proteins in our *P*. *aeruginosa* membrane vesicle preparations is consistent with a heterogeneous population of OMVs and O-IMVs. Outer membrane proteins were significantly more abundant than cytoplasmic or cytoplasmic membrane proteins (p < 0.05), which is consistent with the previous observation that a majority of *P*. *aeruginosa* membrane vesicles are OMVs rather than O-IMVs.

**Table 1:** Subcellular localization of 66 PAO1 OMV core proteins.

Compartments detected in	PAO1 OMV Core Protein
**Cytoplasmic**	AceE, AceF, AcnB, AhpC, ArcA, ArcB, AspS, AtpA, AtpD, CarB, DnaK, FusA1, GroEL, GuaB, HflC, HtpG, HupB, IlvC, Lpd, MqoB, NrdA, NrdB, PA3001, PA4671, PA5046, PpsA, Prs, PyrG, RecA, Rne, RplA, RplK, RplN, RplT, RpoA, RpoB, RpoC, RpsA, RpsC, RpsD, RpsI, SucA, SucC, Tig, TufA
**Cytoplasmic Membrane**	DadA, MexA, MexB, PA1767, PA3729, SdhA
**Periplasmic**	FliC, OprF, RpsA
**Outer Membrane**	Lpd, LptD, MexA, OprD, OprF, OprI, OprM, OprQ, PA0041, PA0641, PA1288, PA2462
**Outer Membrane Vesicle**	AceE, AceF, AcnB, AhpC, ArcA, ArcB, AspS, AtpA, AtpD, CarB, DadA, DnaK, EtfB, FlgE, FliC, FusA1, GroEL, HflC, HtpG, HupB, Idh, Lpd, LptD, MexA, MexB, MqoB, NrdA, OprD, OprF, OprI, OprM, OprQ, PA0641, PA1288, PA1767, PA2462, PA3001, PA3729, PA4671, Prs, RecA, Rne, RplA, RplK, RplN, RplT, RpoA, RpoB, RpoC, RpsA, RpsC, RpsD, RpsI, SdhA, SucA, Tig
**Extracellular**	FlgE, FlgL, FliC, PA0041
**Flagellar**	FlgL, FliC

Subcellular localization annotations were retrieved from Pseudomonas Genome Database (http://www.pseudomonas.com/localizations/list). Proteins can be associated with multiple subcellular localizations.

### Conserved OMV proteins

**Fig 3:**
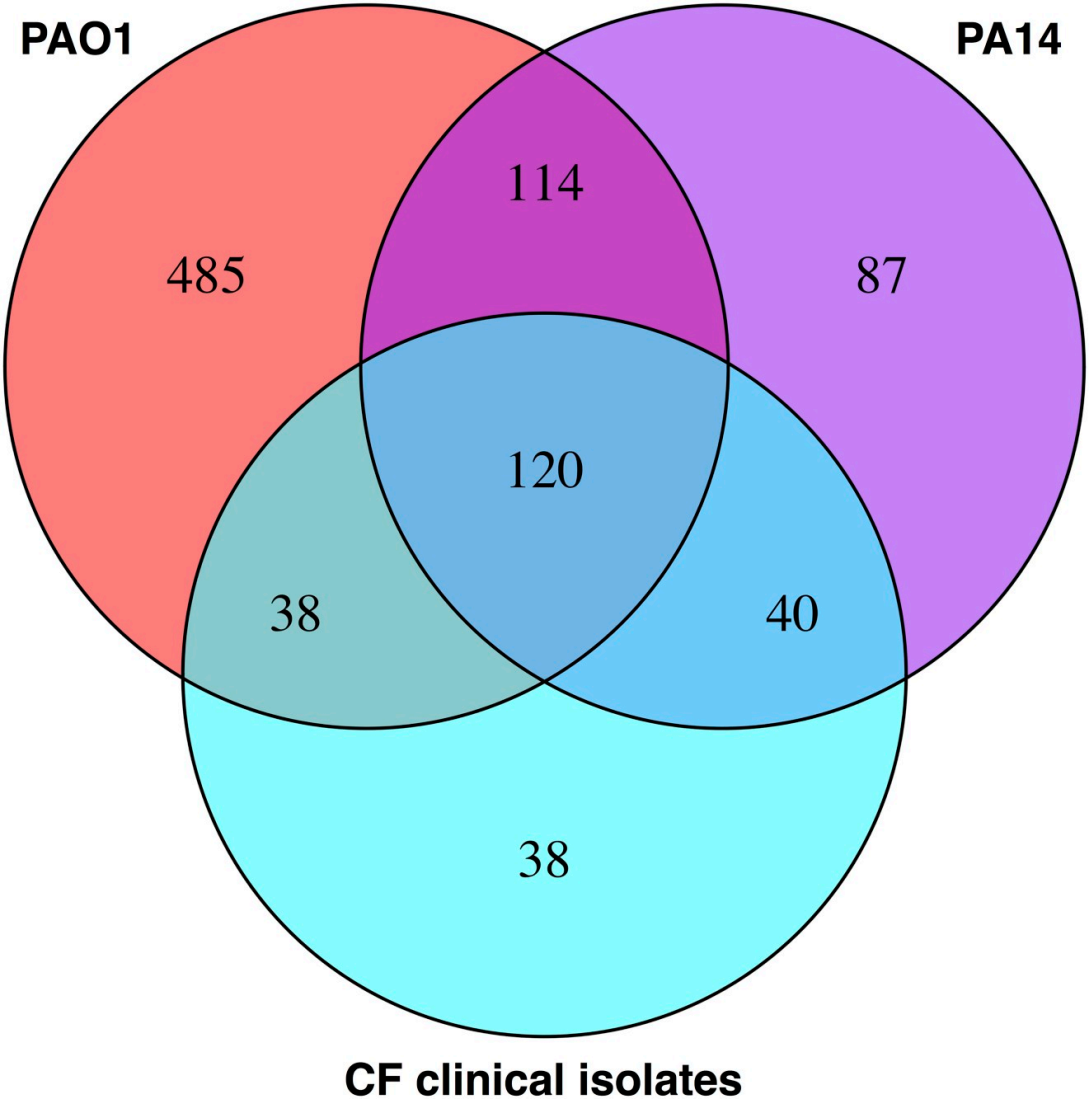
Conserved OMV proteins. 120 proteins were detected in OMVs from *P*. *aeruginosa* strain PAO1 as well as PA14 and two CF clinical isolates [25].

To identify conserved OMV-associated proteins across distantly related strains of *P*. *aeruginosa*, we compared our PAO1 Ctl OMV proteome with the OMV proteome from PA14 and two CF clinical isolates also grown in LB medium [25]. We found that 120 proteins were detected in OMVs from all four strains (Fig 3). Several of these conserved proteins are known to be directly or indirectly involved in virulence or antibiotic resistance, including AprA, CbpD, FliC, FliD, LasA, LasB, MexA, MexB, PepA, PilQ, PlcN, and PrpL/Piv. MexA and MexB are part of an efflux pump conferring antimicrobial resistance and PilQ is involved in the secretion of pilus proteins. The subcellular localization of the 120 proteins conserved in OMVs across different *P*. *aeruginosa* strains is listed in Table 2.

**Table 2:** Subcellular Localization of 120 OMV proteins conserved in PAO1, PA14 and CF clinical isolates.

Compartments detected in	Conserved OMV Proteins
**Cytoplasmic**	AccC, AceE, AceF, AcnB, ArcA, ArcB, AtpA, AtpD, BfrB, CcoO2, CysE, DnaK, FabG, Fda, FolE2, FusA2, GdhB, GroEL, GuaB, HflC, HtpG, LldD, NqrA, NrdA, NuoD, PA3001, PA3309, PA3848, PA4352, PepA, Pfm, PpsA, ProA, PrpC, Prs, PurC, RecA, RplD, RplE, RplJ, RplN, RplP, RplQ, RplR, RplS, RplT, RplU, RplV, RpmB, RpoA, RpoB, RpoC, RpoD, RpsA, RpsB, RpsC, RpsD, RpsG, RpsI, RpsM, RpsQ, RpsU, SecA, SucB, SucC, WbpA
**Cytoplasmic Membrane**	CtpA, DacC, DadA, MexA, MexB, PA2815, PA3734, PA4431, Psd, SdhA, SdhB
**Periplasmic**	AatJ, AnsB, BraC, DacC, FabG, FliC, GlpQ, OpgG, OprF, RpsA
**Outer Membrane**	LasA, LipA, LptD, LptE, MexA, OprB, OprD, OprF, OprG, OprH, OprI, OprL, OprM, OprQ, PA0833, PA1053, PA1288, PA4974, PagL, PilQ
**Outer Membrane Vesicle**	AceE, AceF, AcnB, ArcA, ArcB, AtpA, AtpD, BfrB, BraC, CcoO2, CtpA, DacC, DadA, DnaK, FliC, FliD, FolE2, GdhB, GroEL, HflC, HtpG, Idh, LasA, LldD, LptD, LptE, MexA, MexB, NqrA, NrdA, OprB, OprD, OprF, OprG, OprH, OprI, OprL, OprM, OprQ, PA0537, PA0622, PA0623, PA0833, PA1053, PA1288, PA2815, PA3001, PA3309, PA3848, PA4352, PA4431, PA4639, PA4974, PagL, PasP, PepA, PilQ, Prs, Psd, RecA, RplD, RplE, RplN, RplQ, RplT, RplU, RpoA, RpoB, RpoC, RpoD, RpsA, RpsB, RpsC, RpsD, RpsG, RpsI, SdhA, SdhB, SecA, SucB, WbpA
**Extracellular**	AatJ, AprA, CbpD, FliC, FliD, LasA, LasB, LipA, PA2939, PasP, PepA, Piv, PlcN
**Flagellar**	FliC, FliD
**Unknown**	PA1324, PA2635, PA3922, PA4139, PA4140

Subcellular localization annotations were retrieved from Pseudomonas Genome Database (http://www.pseudomonas.com/localizations/list). Proteins can be associated with multiple subcellular localizations.

### Differential abundance of proteins in Tobi OMVs and Ctl OMVs

The amount of protein in OMV preparations, as determined by Micro BCA Protein Assay, was 294.3 ± 16.6 μg/ml for ctl OMVs and 186.3 ± 8.7 μg/ml for Tobi OMVs. The reduction in protein concentration of Tobi OMVs of -108.0 ± 18.7 μg/ml was statistically significant (p < 0.05, n = 3). The number of OMVs, quantified by Nanoparticle Tracking Analysis, was 2.4*10^11^ ± 7.1*10^10^/ml for ctl OMVs and 1.1*10^11^ ± 4.0*10^10^/ml for Tobi OMVs. The difference in OMV number between groups was not statistically significant. We used the same amount of protein for each sample (20 μg) as input for the proteomics experiment. There was no significant difference between the number of Ctl OMVs and Tobi OMVs needed to obtain 20 μg protein.

**Fig 4:**
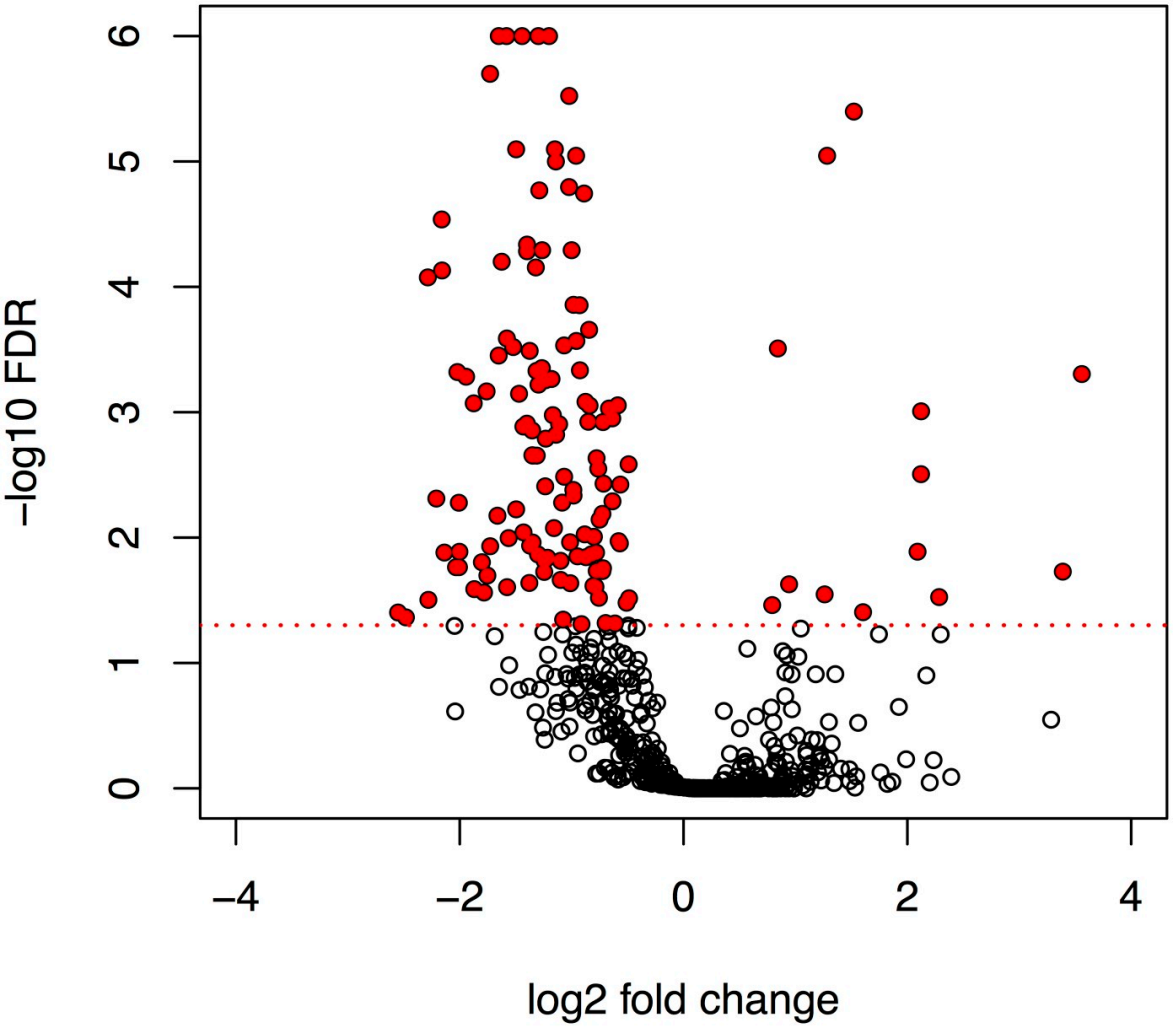
Differential abundance analysis of integrated peak intensities for 761 proteins associated with Tobi OMVs and Ctl OMVs. Volcano plot of log2 fold changes and FDR-corrected p-values obtained with QPROT. 182 proteins with FDR < 0.05 were considered significantly differentially abundant (red circles). 165 proteins were down-regulated in Tobi OMVs while 17 proteins were up-regulated. Horizontal red dotted line indicates FDR = 0.05. Low abundance proteins, defined as proteins detected in fewer than 2 replicate samples, as well as proteins exclusively detected in one of the groups, were not included in the analysis.

Tobramycin significantly decreased the abundance of 165 proteins and increased the abundance of 17 proteins in OMVs (Fig 4 and S2 Table). Among the proteins that were significantly decreased in Tobi OMVs were several known virulence determinants, including AprA, AlpA/D/E, LasI, PlcN, CbrA and several proteins involved in LPS O-antigen biosynthesis and type VI secretion system.

While several of the differentially abundant OMV proteins would be predicted to affect the host response or host-pathogen interaction if they were differentially abundant in whole cell *P*. *aeruginosa*, we did not measure protein changes at the whole cell level. Due to differential packaging of proteins into OMVs, the OMV protein cargo does not reflect whole cell protein levels. Thus, we focus the discussion on differentially abundant virulence factors that have a direct effect on the host immune response. Tobramycin reduced the abundance of AprA in OMVs by about 2-fold. High levels of AprA are associated with the mucoid phenotype and clinical exacerbations in CF [56, 57]. AprA, an alkaline protease, enhances *P*. *aeruginosa* survival in the lung by proteolytically activating ENaC, thereby increasing sodium reabsorption, an effect that dehydrates the airway and decrease mucociliary clearance of bacteria [58, 59]. Increased sodium reabsorption by ENaC also causes membrane depolarization and reduces the electrochemical gradient for CFTR Cl^-^ secretion, resulting in further airway dehydration. Thus, a Tobramycin-mediated decrease in AprA delivery to airway cells by OMVs is predicted to reduce airway dehydration and enhance clearance of *P*. *aeruginosa* from the lungs.

Tobramycin also reduced the abundance of AlpA, AlpD and AlpE in OMVs. *P*. *aeruginosa* deletion mutants for AlpA and AlpBCDE have attenuated virulence and reduced lung colonization in a murine infection model [19]. Hence, a Tobramycin-mediated reduction of AlpD and AlpE in OMVs may reduce *P*. *aeruginosa* self-lysis and thus decrease lung damage and improve lung function.

A previous study did not report any effect of 1 μg/ml Tobramycin on the protein levels of AprA or AlpA/D/E in whole cell *P*. *aeruginosa* [60], suggesting that these proteins are selectively packaged into OMVs.

Among the differentially abundant OMV-associated proteins with the largest fold changes in response to Tobramycin are many uncharacterized hypothetical proteins (S2 Table). Future studies are needed to assess the function of these proteins and the effect they may have on the host. Seven proteins were among the top 10 most abundant proteins in both Ctl and Tobi OMVs: PA0622, PA1053, FliC, OprH, PA2939, FabZ and PA3734. OprQ, OprI and Ssb were among the 10 most abundant proteins in Ctl, but not Tobi OMVs, while OprF, PA4141 and RplN were among the top 10 in Tobi but not Ctl OMVs.

Finally, we looked for proteins that were exclusively detected in either Ctl or Tobi OMVs and found that 53 proteins were reliably detected in all replicates of Ctl OMVs, but not Tobi OMVs, while 4 proteins were detected exclusively in Tobi OMVs (S3 Table).

### Phe508del CFTR Cl^-^ secretion

**Fig 5:**
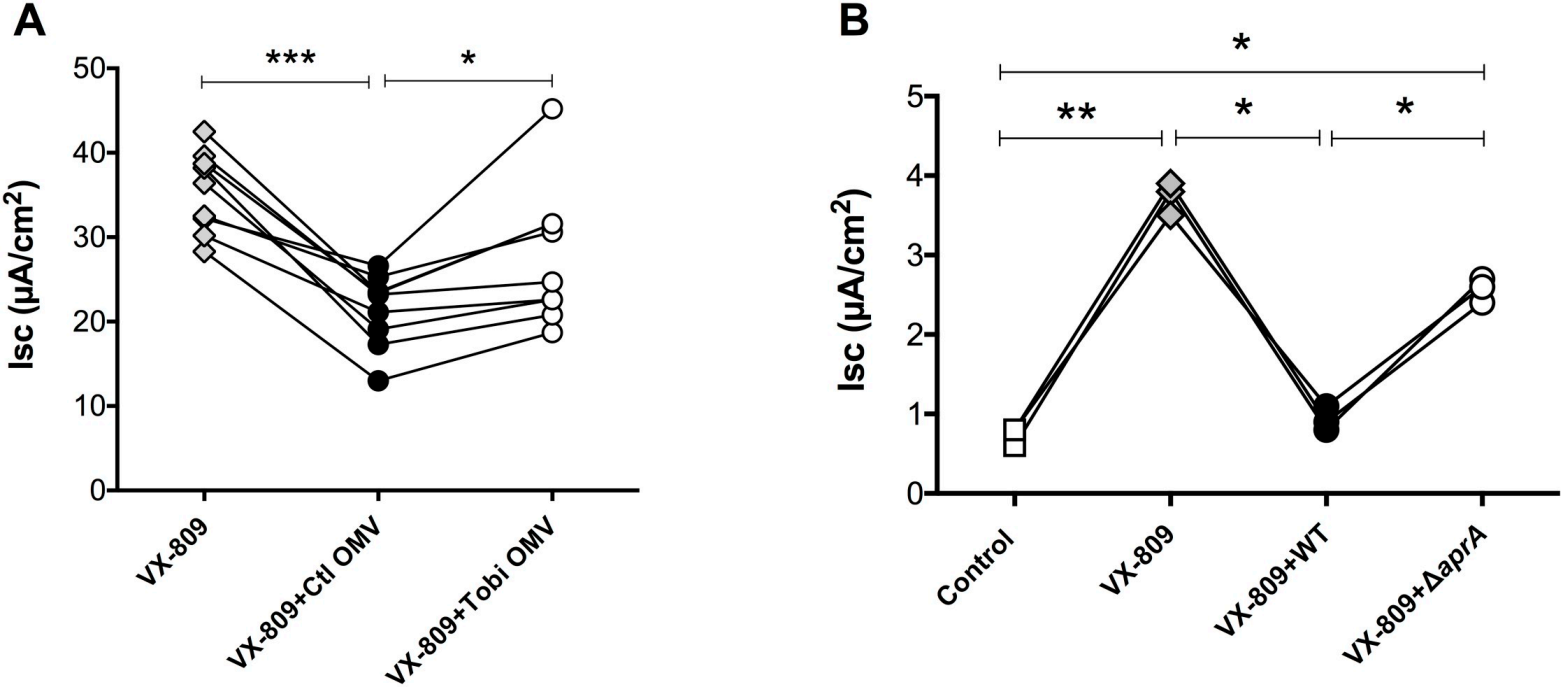
Phe508del-CFTR Cl^-^ secretion. **(**A) CFBE cells were treated with VX-809 (3 μM for 48 h) and exposed to vehicle, Ctl OMVs or OMVs isolated from Tobramycin treated *P*. *aeruginosa* (Tobi OMV) for 1.5 h prior to measurements of Phe508del CFTR Cl^-^ secretion (presented as *μ*A/cm^2^). Ctl OMVs reduced VX-809 Phe508del CFTR Cl^-^ secretion compared to VX-809 alone (P<0.001). Phe508del CFTR Cl^-^ secretion in the VX-809+Tobi OMV treated cells was significantly higher than in the VX-809+Ctl OMV treated cells (P<0.05), while OMVs isolated from *P*. *aeruginosa* treated with Tobramycin did not significantly reduce VX-809 Phe508del CFTR Cl^-^ currents compared to VX-809 alone. (B) CF-HBE cells were treated with vehicle (Control), VX-809 alone (3 μM for 48 h), or VX-809 and PA14 wild type (WT) or VX-809 and PA14-Δ*aprA* for 6 h prior to measurements of Phe508del CFTR Cl^-^ secretion. VX-809 increase Phe508del CFTR Cl^-^ secretion (P<0.01). PA14 eliminated the VX-809 stimulated Phe508del CFTR Cl^-^ secretion (VX-809 versus VX-809+ PA14, P<0.05). The PA14 Δ*aprA* deletion mutant did not reduce Phe508del CFTR Cl^-^ secretion as much as PA14 (P<0.05). *** P<0.001, **P<0.01, *P<0.05.

Characterization of the proteome of OMVs isolated from control and Tobramycin-exposed *P*. *aeruginosa* revealed that Tobramycin reduced AprA, which is predicted to mitigate the inhibitory effect of *P*. *aeruginosa* on Phe508del-CFTR Cl^-^ secretion. In previous studies, we reported that OMVs inhibit both wt-CFTR and VX-809 stimulated PheF508-CFTR Cl^-^ secretion [7, 9]. To validate the biological significance of the Tobramycin-induced reduction in AprA, we conducted two sets of experiments. First, as predicted by our proteomic analysis, OMVs isolated from Tobramycin treated *P*. *aeruginosa* had a less inhibitory effect on Phe508del-CFTR Cl^-^ secretion by CFBE cells than OMVs isolated from control *P*. *aeruginosa* (Fig 5A). Second, deletion of the *aprA* gene reduced the inhibitory effect of *P*. *aeruginosa* on Phe508del-CFTR Cl^-^ secretion by CF-HBE cells (Fig 5B).

## Conclusions

The goal of this study was to evaluate the effect of Tobramycin on the proteome of OMVs secreted by *P*. *aeruginosa*. There are several limitations to the present study. We only examined the effect of Tobramycin on one strain of *P*. *aeruginosa*, PAO1, at one concentration and in one growth medium. Previous studies have shown that gene and protein expression by *P*. *aeruginosa* is dependent on strain, growth conditions and nutrient availability [47–50]. Additional studies, beyond the scope of the present report, are required to evaluate the effect of these factors on Tobramycin regulation of virulence factor expression in OMVs. Despite these limitations, to our knowledge our study is the first to examine the effect of Tobramycin on the proteome of OMVs secreted by *P*. *aeruginosa*. Tobramycin reduced the abundance of several OMV-associated virulence determinants, most notably AprA and AlpA/D/E. AprA was in the conserved OMV proteome of PAO1, PA14 and two clinical isolates of *P*. *aeruginosa* (Table 2). Functional validation of the proteomic data confirmed that the Tobramycin-induced decrease in AprA virulence factor in OMVs mitigates the inhibitory effect of OMVs on Phe508del CFTR Cl^-^ secretion by VX-809 stimulated CF bronchial epithelial cells (Fig 5). Taken together, our proteomic analysis of OMVs and biological validation suggest that Tobramycin may improve lung function in CF patients infected with *P*. *aeruginosa* in part by reducing AprA in OMVs, an effect that would mitigate the adverse effect of OMVs on Phe508del CFTR Cl^-^ secretion, which is essential for bacterial clearance from the lungs.

In conclusion, we suggest that the Tobramycin-induced reduction in AprA, AlpA, AlpD, AlpE, and other virulence determinants in OMVs may reduce lung damage and improve lung function, thereby providing a positive clinical benefit with only a modest reduction in bacterial load.

## Supporting information

S1 Table Raw and normalized proteomic data. (XLSX)

S2 Table Differentially abundant proteins. (XLSX)

S3 Table Proteins exclusively identified in Ctl or Tobi OMVs. (XLSX)
